# Radiation Proctitis: A Review of Pathophysiology and Treatment Strategies

**DOI:** 10.7759/cureus.70581

**Published:** 2024-09-30

**Authors:** Mohit Bhatia, Hadeel Suliman, Rizwan Ahmed, Danko Kostadinov, Tarun Singhal

**Affiliations:** 1 Colorectal and General Surgery, Princess Royal University Hospital, King's College Hospital NHS Foundation Trust, London, GBR; 2 General Surgery, Princess Royal University Hospital, King's College Hospital NHS Foundation Trust, London, GBR

**Keywords:** cancer radiotherapy, pelvic malignancy, radiation, radiation proctitis, severe proctitis

## Abstract

Radiotherapy (RT) has become an integral part of cancer treatment worldwide; it aims to arrest the uncontrolled growth of tumor cells by using high-energy rays. Radiation proctitis is a known clinical manifestation after the RT regime for pelvic malignancies. Radiation proctitis can have a variable presentation, and there are a lot of patient-related factors that can affect the eventual outcome. In most instances, it is self-limiting; however, it can become chronic in some cases and can affect the quality of life. Many treatment options are recommended, but there has been no consensus on the treatment protocols for managing this known clinical condition. We have tried to briefly describe its pathogenesis, important factors affecting the outcome, and available treatment strategies.

## Introduction and background

Radiotherapy(RT) is an important treatment modality in practice for various malignancies. Its main principle is using high-energy rays targeted at diseased tissue to restrict the growth of rapidly dividing cells [1].

In 1895, Rontgen highlighted the use of X-rays, and subsequently, its use in treating cancers was studied and adopted in practice. Krause and Ziegler studied the harmful effects of radiation on the adjacent normal tissues; their main interest of study was the harmful effects of radiation on the small bowel [2].

In 1930, Buie described the effects of radiation therapy, resulting in proctitis in patients who were subjected to pelvic irradiation [3].

RT is believed to cause deoxyribonucleic acid (DNA) damage, which affects the lipid/protein core and eventually leads to apoptosis and cellular death. Radiation proctitis (RP) is a known complication after radiation therapy in pelvic malignancies, especially in patients with prostatic cancer [4].

Pelvic radiation therapy is widely used in treating prostatic cancers, lower gastrointestinal cancers, and gynecological malignancies. Based on the merit of the case, its use can be neo-adjuvant or adjuvant. Owing to its proximity to the target organs and fixed anatomy, the rectum is highly vulnerable for developing radiation-induced injury [5].

It is believed that around 75% of the patients receiving conventional pelvic RT eventually suffer from RP. Most of the time, it is self-limiting; however, in certain cases, it can become a chronic issue with refractory symptoms. RP is characterized by damage to rectal epithelium due to ionizing radiation therapy [6]. Chronic RP involves full thickness of the rectal mucosa and results in fibrosis and obliterative arteritis [7].

## Review

Pathophysiology

**Figure 1:**
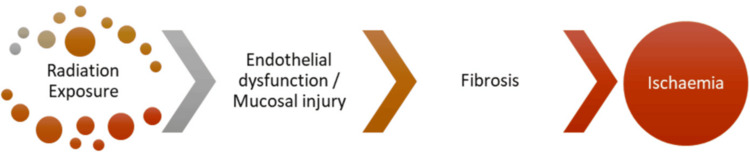
Steps in the development of proctitis Ref. [7]

Figure 1 shows the steps in the pathophysiology of radiation proctitis.

Types

Acute RP

This subtype usually manifests in the immediate period post-radiation therapy but can appear first up to three months post-radiation. This involves superficial mucosa, and its presentation can be variable; however, the most commonly presenting features include bleeding per rectum, diarrhea, cramps, mucus discharge, and urgency [8].

Chronic RP

Its presentation symptoms can occur or persist for many months to years even after completion of radiation therapy. It mostly manifests as rectal bleeding and refractory discomfort and can eventually lead to stricture or perforation. These changes are believed to occur due to a compromise in the blood supply, which results in chronic fibrotic changes in the rectal wall [1]. It is estimated that 29-89.6% of patients can have refractory rectal bleeding post-irradiation [9]. Long-standing symptoms in the chronic phase can affect the quality of life [10].

For the diagnosis of RP, clinical history and examination can aid in getting to a diagnosis. Fecal calprotectin and lactoferrin can be used for evaluating cases of RP; however, their use is not widely practiced. Their use is based on the principle of intestinal inflammation caused by migrating neutrophils. Studies have shown that calprotectin and lactoferrin levels are increased due to radiation-induced injury changes [11].

Endoscopic Diagnosis

Endoscopic evaluation plays an integral role in diagnosing RP. Colonoscopy or flexible sigmoidoscopy can be used and often helps in excluding other causes that may mimic the clinical presentation of RP. Pathognomonic signs of RP on endoscopy include friable mucosa, bleeding, ulceration, telangiectasia, pallor, and ulceration [12].

In chronic RP, endoscopy reveals mucosal atrophy, pale mucosa with tortuous blood vessels, and fibrotic strictures [13]. The grading of proctitis is shown in Table 1.

**Table 1:** Grading of radiotherapy (RP) Ref. [14]

Grades	Description
0	No symptoms
1	Mild diarrhea, mild cramping, bowel movements 5 times daily, slight rectal discharge
2	Moderate diarrhea and colic bowel movement >5 times a day, excessive rectal mucus or bleeding
3	Obstruction or bleeding requiring surgery
4	Necrosis/perforation/fistula

Factors Affections the Outcome

Dose/duration/delivery: It is believed that the incidence of developing RP is dependent on the duration, dose, and delivery method of RT. Some studies suggest the type of tissue getting irradiated also can play a role in being susceptible to developing the aftermaths of radiation [15]. Studies have shown radiation doses less than 45 gray (Gy) are usually associated with a low incidence of side effects, whereas this incidence is high in individuals receiving doses greater than (>70 Gy). Doses above 70 Gy have a higher chance of causing injury to the surrounding area and tissue [16].

It is believed that external beam radiation therapy (EBRT) is associated with a higher incidence of RP, around 2-39%. This incidence is higher than those noted in the case of particle radiation therapy or intensity-modulated radiation therapy (IMRT). Therefore, it is recommended that patients receiving EBRT should have an intermittent break in radiation therapy to decrease the irradiation effects [17]. It is observed that patients receiving brachytherapy usually encounter less severity of side effects as compared to EBRT [16].

Patients who receive a combined chemotherapy and radiotherapy regimen are more likely to have intestinal symptoms post-radiotherapy [18]. A retrospective analysis showed a reduction in the incidence of rectal toxicity in patients who received image-guided IMRT as compared to the ones without image guidance [19].

In patients suffering from HIV, there is a depletion of radioactive thiols, which can enhance the risk of radiation-induced mucositis and bleeding [20]. Comorbidities, including diabetes, hypertension, collagen vascular disease, vasculitis, and atherosclerosis, are believed to enhance radiation injury [16]. Some studies have shown that the presence of hemorrhoids and anticoagulant use can increase susceptibility to radiation-induced proctitis [21].

**Figure 2:**
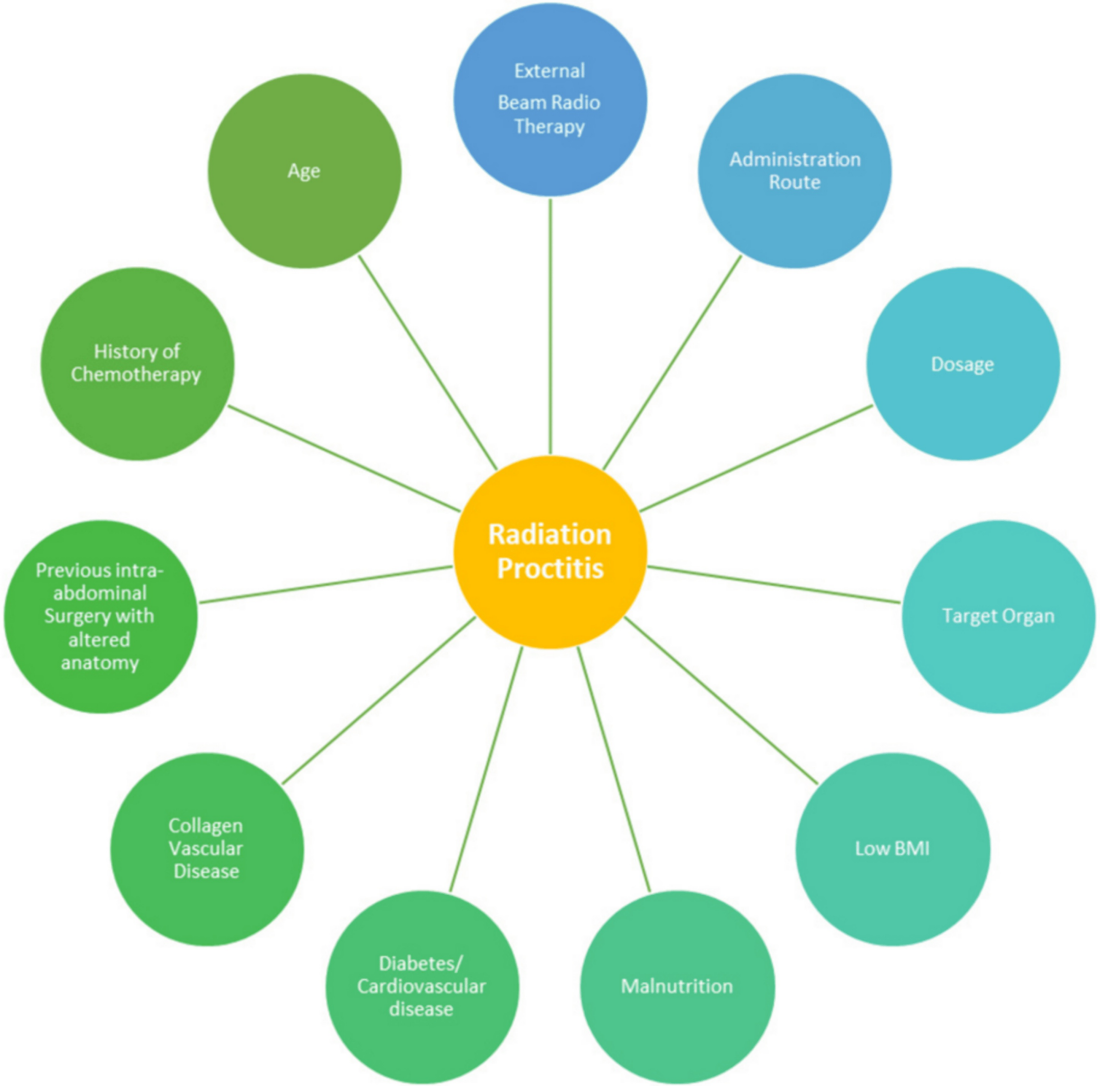
Factors increasing the incidence of developing radiation proctitis Ref. [7]

Factors affecting the outcome and severity of RP are shown in Figure 2.

Treatment

Acute RP is mostly self-limiting in the majority of cases; therefore, it can be managed with symptom control by using adequate hydration, steroid enema, and anti-diarrhoeal medications [19].

Various medications have been used in clinical practice to resolve the symptoms of RP.

Formalin

Formalin-soaked gauze is believed to result in chemical cauterization when directly applied to the affected mucosa, which helps in achieving hemostasis. Usually, contact for two to three minutes is considered sufficient to enable its action on the mucosa [22]. In 1969, Brown highlighted the usefulness of formalin in treating proctopathy associated with radiation therapy [23]. Its use is generally considered safe; however, complications such as anorectal pain, rectosigmoid necrosis, and strictures have been reported [24].

Biswal et al. [25] showed effectiveness in around 81% of patients with radiation proctopathy treated with formalin. A study suggested the safest and most effective way of applying formalin under direct visualization using sigmoidoscopy. They believed that, with direct visualization, the chances of the target area being precisely located would be reduced, reducing the chances of complications [26].

Raman et al. [27] used a lower concentration of formalin (2%) and showed positive results with a low incidence of complications. Stern reported two cases of anorectal cancer after the use of formalin therapy; however, there was no direct relationship between formalin and anorectal cancer [28]. Some authors have compared formalin versus colonic irrigation with oral antibiotics and found superior results and satisfaction among radiation proctopathy patients [29].

Steroids

Due to the anti-inflammatory action, steroids have been used in managing RP. They can be used along with other medications such as metronidazole to alleviate symptoms such as rectal bleeding and diarrhea [6]. Cavcic et al. [30] suggested that metronidazole has a synergistic effect when used along with steroidal formulations in treating chronic RP. Takemoto et al. [31] reported 62% efficacy in treating RP using steroid enemas. They suggested steroid enemas help in stabilizing the membrane and reduce the incidence of rectal bleeding.

A randomized trial showed a higher incidence of bleeding and mucus discharge per rectum after treatment with hydrocortisone foam [32].

Anti-inflammatory Drugs

Sulfasalazine (mesalamine) works by inhibiting prostaglandin synthesis and helps in reducing free radical scavenging activity. Kochar et al. showed that patients responded better with sucralfate alone compared to the group receiving sulfasalazine with rectal steroids [33].

Due to its proven effectiveness in treating ulcerative colitis, its role was studied in treating radiation proctitis. In both RP and inflammatory bowel disease, there is an inflammatory process that results in an increase in interleukin 2 and 6. Due to the anti-inflammatory action of mesalamine, it was used to study its role in resolving RP [34].

Jahraus et al. [35] studied the role of balsalazide (a derivative of 5-aminosalicylic acid (ASA)) in treating radiation proctopathy. This study showed satisfactory results with few side effects. Anti-inflammatory drugs derived from 5-ASA work by reducing the free radicals and inhibiting prostaglandin synthesis [36]. Wu et al. [37] suggested that a 0.5-g mesalazine suppository is effective in refractory rectal ulceration post-radiation therapy.

Sucralfate

It is believed to form a protective epithelial barrier that enables healing of the epithelium. Rectal sucralfate (3 g) has shown superior results in radiation proctitis compared with anti-inflammatory medications [38]. Few authors suggested using sucralfate as a paste formulation results in better local application results in treating proctitis [39].

A study showed resolution in symptoms such as diarrhea and urgency in patients treated with sucralfate when compared with the placebo group [40]. Though the exact mechanism of its function is not known, it is believed that, due to its angiogenic action, it helps increase the local blood flow, exerts cytoprotective action, and enhances mucosal repair [41]. It can form a protective barrier at the injury site and can reduce the damage caused by the enzymes and defecation [42].

Short-Chain Fatty Acids (SCFA)

SCFA is normally synthesized by colonic bacteria. It is believed to enhance mucosal blood flow due to its vasodilatory action, which can enhance healing [43]. Some trials observed that SCFA may have a resolving effect on ARP, but its role in treating long-term proctitis is debatable [44]. Another randomized trial showed superior results in symptom relief and healing compared with SCFA enema when compared to the placebo group [45].

Argon Plasma Coagulation (APC)

Endoscopic APC helps in resolving symptoms evidently in 70-100% of patients with chronic RP. It involves using high-frequency energy without point contact and utilizing argon gas to result in coagulation, which helps in controlling bleeding due to proctitis [46].

A randomized trial showed superior results of APC in resolving rectal bleeding but was not found to be effective in resolving other symptoms due to CRP [47]. There is no consensus yet regarding the number of sessions required for treating the bleeding; a study by Higuera et al. [48] observed 1.6 as the mean number of sessions; contrary to these results, a study by Sudha et al. [49] reflected a mean of five days to successfully treat rectal bleeding.

There is a debate about whether the severity of proctitis dictates the success of treating with APC. Some authors have postulated that the degree of proctitis does not alter the treatment outcome with APC. They also used the total colonoscopic severity score (TCSS) to determine the effectiveness of APC in treating RP. These scoring systems have not been standardized, and therefore the results can be variable [50].

Some reported side effects of APC include abdominal distension due to argon insufflation, ulceration, and perforation [51]. Lenz et al. reported a 7% risk of developing bacteremia post-APC therapy [52]. Some authors have suggested an increased incidence of rectal ulcers associated with the intake of non-steroidal anti-inflammatory drugs (NSAIDs); therefore, their use should be very limited, especially when subject to APC therapy [53].

Though there are no large studies reflecting the effectiveness of APC, there are numerous small studies that have shown encouraging results with the use of APC in resolving RP, and some studies have shown remission rates around 90% in treating radiation proctopathy [54].

The yttrium aluminum garnet (YAG) laser also works on the principle of non-contact thermoregulation. This works best at deeper tissue level as compared with the APC. Its application is believed to be more difficult and is usually associated with stenosis, necrosis, and fistula formation [55].

Hyperbaric Oxygen (HO)

HO therapy involves using intermittent inhalation of 100% oxygen in a chamber with pressure higher than the atmospheric pressure [51]. Radiation-induced damage includes occlusive endarteritis and mucosal damage; therefore, with the use of HO, there is a higher chance of neo-vascularization and collagen formation, which can promote healing [56].

The American Society of Colon and Rectal Surgeons also recommends the use of HO therapy in chronic RP. Their recommendation is based on the angiogenic effects, which improve tissue oxygenation [57].

The HO therapy regime usually includes 60-120 minutes of therapy, and sessions can be variable and tapered depending on the response and patient tolerance [58]. HO therapy should not be used as a first-line treatment but rather should be considered in refractory RP where other first-line therapies have failed [59].

Charneau et al. [60] treated the first patient of RP with HO therapy in early 1991. Most of the studies involving the usefulness of HO therapy have been small sample studies; however, Tahir et al. found a 95% response rate in 59 patients [61].

Surgery

As discussed before, most RP episodes are self-limiting or satisfactorily managed with medical management; however, some patients with refractory symptoms or the ones affected by severe RP, which hampers their quality of life, are usually candidates for surgical intervention [62]. Around 10% of the RP patients eventually need surgical intervention, as per the literature [63]. Fecal diversion (ostomy) is the most often performed surgical intervention for severe or refractory RP. With the diversion of the fecal route, symptoms such as tenesmus, incontinence, and bleeding are resolved to some extent. Resection and anastomosis are not considered a feasible option due to the damaged tissue and the affected microcirculation, which can hamper the healing and rather result in further complications [64].

Diversion of colostomies can help in reducing bacterial contamination and can prevent ulcers resulting in perforation. Some studies have shown superior results with transverse colostomy over loop colostomy due to its chances of easy reversal and preservation of superior rectal and marginal vessels [65].

A large study in their results depicted that the transverse colostomy helped in reducing the incidence of severe bleeding associated with RP, and they recommended it as a treatment modality when dealing with severe bleeding or refractory chronic RP [66]. Of course, creating a stoma can have implications on both the physical and mental aspects of the patient; therefore, the risk versus benefit should be assessed by the primary clinician, and a large study size in the future should be able to highlight better results. 

**Figure 3:**
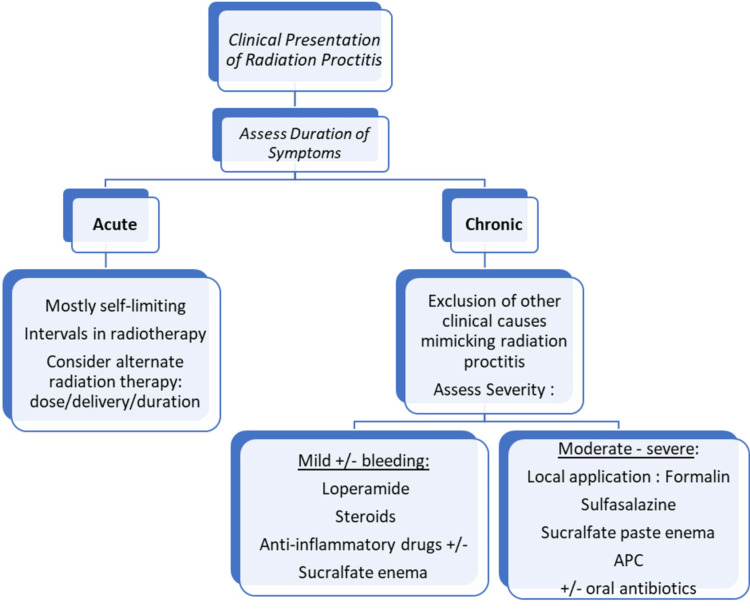
Flowchart for the proposed planning treatment in RP

Figure 3 shows the proposed treatment algorithm for RP.

## Conclusions

RP is a known clinical condition after a radiotherapy regime that can present with variable symptoms and can eventually impact quality of life. Therefore, proper planning and a multidisciplinary approach should be used before finalizing the treatment protocols. Patients should be made aware of the possible implications and should be clinically assessed after RT sessions with an open-minded approach. The patient’s age, associated comorbidities, performance status, severity of proctitis, and patient expectations are some important factors to consider while planning treatment. There are different treatment protocols for treating RP; however, there is no generalized consensus yet. The treating clinician should follow the institutional protocols and should always exclude other causes mimicking RP before starting the treatment regime.
